# Comparing a global high-resolution downscaled fossil fuel CO_2_ emission dataset to local inventory-based estimates over 14 global cities

**DOI:** 10.1186/s13021-020-00146-3

**Published:** 2020-05-19

**Authors:** Jingwen Chen, Fang Zhao, Ning Zeng, Tomohiro Oda

**Affiliations:** 1 State Key Laboratory of Hydrology-Water Resources and Hydraulic Engineering, Hohai University, Nanjing, 210098 China; 2 College of Hydrology and Water Resources, Hohai University, Nanjing, 210098 China; 3 School of Geographical Sciences, East China Normal University, Shanghai, 200241 China; 4 Key Laboratory of Geographic Information Sciences (Ministry of Education of China), East China Normal University, Shanghai, 200241 China; 5 Potsdam Institute for Climate Impact Research, Potsdam, 14473 Germany; 6 Department of Atmospheric and Oceanic Science, University of Maryland, College Park, MD USA; 7 Earth System Science Interdisciplinary Centre (ESSIC), University of Maryland, College Park, MD USA; 8 Global Modeling and Assimilation Office (GMAO), NASA’s Goddard Space Flight Centre (GSFC), Greenbelt, MD USA; 9 Goddard Earth Sciences Research and Technology, Universities Space Research Association, Columbia, MD USA

**Keywords:** Fossil fuel CO_2_ emissions, Emission inventory, ODIAC, City CO_2_ emissions, In-boundary

## Abstract

**Background:**

Compilation of emission inventories (EIs) for cities is a whole new challenge to assess the subnational climate mitigation effort under the Paris Climate Agreement. Some cities have started compiling EIs, often following a global community protocol. However, EIs are often difficult to systematically examine because of the ways they were compiled (data collection and emission calculation) and reported (sector definition and direct vs consumption). In addition, such EI estimates are not readily applicable to objective evaluation using modeling and observations due to the lack of spatial emission extents. City emission estimates used in the science community are often based on downscaled gridded EIs, while the accuracy of the downscaled emissions at city level is not fully assessed.

**Results:**

This study attempts to assess the utility of the downscaled emissions at city level. We collected EIs from 14 major global cities and compare them to the estimates from a global high-resolution fossil fuel CO_2_ emission data product (ODIAC) commonly used in the science research community. We made necessary adjustments to the estimates to make our comparison as reasonable as possible. We found that the two methods produce very close area-wide emission estimates for Shanghai and Delhi (< 10% difference), and reach good consistency in half of the cities examined (< 30% difference). The ODIAC dataset exhibits a much higher emission compared to inventory estimates in Cape Town (+ 148%), Sao Paulo (+ 43%) and Beijing (+ 40%), possibly related to poor correlation between nightlight intensity with human activity, such as the high-emission and low-lighting industrial parks in developing countries. On the other hand, ODIAC shows lower estimates in Manhattan (− 62%), New York City (− 45%), Washington D.C. (− 42%) and Toronto (− 33%), all located in North America, which may be attributable to an underestimation of residential emissions from heating in ODIAC’s nightlight-based approach, and an overestimation of emission from ground transportation in registered vehicles statistics of inventory estimates.

**Conclusions:**

The relatively good agreement suggests that the ODIAC data product could potentially be used as a first source for prior estimate of city-level CO_2_ emission, which is valuable for atmosphere CO_2_ inversion modeling and comparing with satellite CO_2_ observations. Our compilation of in-boundary emission estimates for 14 cities contributes towards establishing an accurate inventory in-boundary global city carbon emission dataset, necessary for accountable local climate mitigation policies in the future.

## Background

Currently 55% of the global population lives in cities in 2018, and the proportion will be 68% by 2050 [1]. With a mere 1% of global land area, cities consume 240EJ energy yearly, and are projected to consume 500–1000EJ in 2100 [2, 3]. Most of this energy is from burning fossil fuels, emitting large quantities of CO_2_ that continues to warm the planet. Therefore, constructing effective plans to reduce emission in cities is a top priority at present. Many cities around the world have actively committed to reduce CO_2_ emission, often in the form of coordinated efforts such as the network of C40 cities (https://www.c40.org/) and the global covenant of mayors for climate and energy (https://www.globalcovenantofmayors.org/).

A key demand for the science community is to accurately monitor changes of greenhouse gas (GHG) emission. It would be the foundation to directly gauge the effectiveness in various mitigation policies implemented by cities and regions worldwide. In addition, such science-based GHG monitoring will support global climate change negotiations to assure the accuracy of reported emissions [4]. Focused efforts on estimating city CO_2_ emission flux has been carried out for some cities, e.g. Megacities over Los Angeles [5], INFLUX over Indianapolis [6], and CO_2_-Megaparis over Paris [7]. These city-scale projects usually require multiple CO_2_ towers and/or flight campaigns to constrain spatial flux distribution of the city [8], and mesoscale atmospheric inversion modeling is often employed to derive spatial flux distribution from CO_2_ concentration measurements and wind. They provide much needed data for top-down estimate of city emissions, however, the labor and economic cost of such projects could be prohibitively high to scale up to a large number of additional cities for long-term observations, especially for megacities in developing countries whose role would be critical in global climate mitigation efforts.

Top-down city emission estimates could potentially be bolstered by the recent development and deployment of carbon observing satellites from the United States, European Union, Japan and China, including NASA’s Orbiting Carbon Observatory 2 (OCO-2) satellite. These satellites show promising aspects of directly measuring the spatiotemporal regional changes of CO_2_ concentrations [9, 10]. For example, OCO-2 is shown to be able to differentiate CO_2_ concentrations at very high temporal and spatial resolution [11], more suitable for local source emission estimations than GOSAT [12], and it has been applied to estimate emission at city scale [13, 14]. While OCO-2 has some limitations on city scale including its spatial and temporal coverage, other existing and planned carbon monitoring satellites such as TanSat (in orbit since 2017), OCO-3 (in orbit since 2019) and GeoCarbon (expected 2023) could potentially enable estimation of CO_2_ emission in an unprecedented number of cities worldwide. Through atmospheric inversion methods, there is great potential to provide an independent estimation on the changes in fossil fuel emission at high spatial resolution based on satellite data, greatly supplementing the sparse flux tower measurements on the ground.

**Fig. 1:**
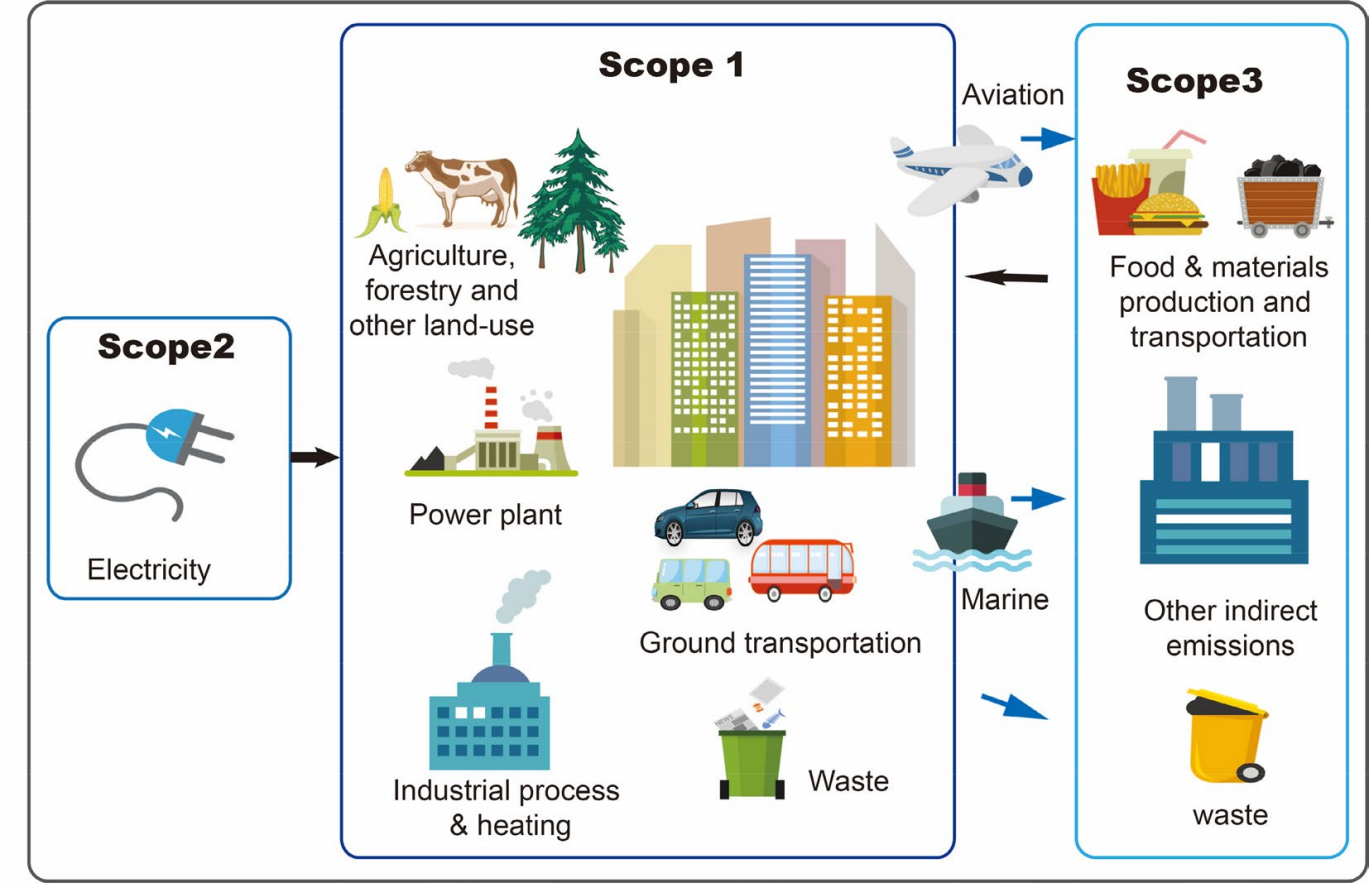
Schematic diagram: the three scopes of city emission. Only scope 1 emissions occurred within city boundary are comparable with inversion results based on observations

In the meantime, cities have started to collect emission inventory data that contribute to bottom-up estimates of emissions, which are usually derived based on various emission activity data such as transportation, electricity generation, industry, and heating. Bottom-up estimates are relatively accurate at global level, and at national level for developed countries [15]. At the city scale, although emission statistics have been reported for some cities around the world [16–20], in many developing countries the statistical infrastructure required for bottom-up inventory is still lacking. Additionally, comparing existing city emission statistics with top-down estimates could be difficult, because the city emission statistics are often not reported in a way that is directly comparable to top-down estimates. An exception is the Hestia data product [21], which provides spatiotemporally-resolved bottom-up estimate of fossil fuel CO_2_ emissions. However, the Hestia approach requires lengthy development time, such that the data is only limited to four U.S. cities [22]. The World Business Council for Sustainable Development and World Resources Institute has defined three different scopes for fossil fuel emissions from different cities [17, 23, 24]. Scope 1 refers to direct emissions produced in city boundaries mainly from fossil fuel combustion, transportation, industrial processes and production, land use and waste. Scope 2 emissions are associated with total electricity consumed in cities and regions generated in and out-of-boundary. Scope 3 include GHG attributable to cities emitted out of city boundaries such as emissions generated during a life-cycle process of production chain, upstream emissions from power plants and out-of-boundary aviation and marine emissions (Fig. 1). For the carbon monitoring community, the most relevant scope is scope 1, which represents direct emission within city boundaries, because the spatial information for scope 2 and 3 emissions is not clear. Therefore, satellite-based estimates cannot track and be compared with the out-of-boundary part of scope 2 and 3 emissions for cities. However, the currently available city-scale inventory estimates are mostly for scope 2 and 3. Due to the nature of data collection (for example, electricity bills do not differentiate whether power is generated within cities), we do not yet have a global base of scope 1 emission in large cities over the globe. The pressing need of estimating direct city emissions thus motivates us to derive in-boundary emissions for some of world’s major cities based on reported inventory statistics in this study.

The top-down and bottom-up estimates of city emissions could potentially complement each other, and they are linked by atmospheric transport modeling [25, 26]. One of the connecting points between them is gridded emission inventories, which resolve the spatial pattern of CO_2_ emission within cities through spatial disaggregation of national emission statistics based on various proxies (population, GDP, nighttime light intensity etc.) [27]. Several gridded CO_2_ emission estimates exist at different spatial resolution and coverage and have served as prior for atmospheric CO_2_ modeling in some case studies: CDIAC with global scale and 1° resolution [28], EDGAR [29] and FFDAS [30, 31] with global scale and 0.1° resolution, and Vulcan [32] with 0.1° resolution only covering the United States [33]. Only the Open source Data Inventory of Anthropogenic CO_2_ emissions (ODIAC) dataset has both global coverage and 1 km resolution [34–37], making it suitable to represent the spatial emission pattern at city scale and potentially comparable to top-down and bottom-up CO_2_ emission estimates over many cities worldwide. In fact, ODIAC data product have been successfully used for CO_2_ analyses at city scales [14, 25, 38, 39]. For the carbon monitoring community, it is then important to understand the potential downscaling bias and uncertainty of the ODIAC emission dataset in different cities in order to obtain robust results and make the estimates more policy relevant.

The focus of our study is to evaluate the downscaling bias and error of the ODIAC dataset over a number of large cities/metropolitan regions, by comparing with in-boundary emission derived from existing inventory city CO_2_ emission estimates. “Data and methods” section describes the ODIAC dataset, boundaries of cities studied, and our methods and assumptions in deriving in-boundary CO_2_ emission based on inventory estimates reported in literature. “Results” section shows the results of comparing the two sources of city emission estimates. “Discussions” and Conclusions sections present discussions and conclusions, respectively.

## Data and methods

### City inventory estimates

In this study, we first retrieved from literature [16, 17, 23, 40–42] inventory emissions for 14 cities and metropolitan areas with various economic status and climate regime across the world (Sao Paulo for the year 2011; Beijing, Shanghai, and Tokyo for the year 2006; Bangkok, Cape Town, Greater Paris, New York City, Manhattan, and Greater Toronto for the year 2005; Greater London for the year 2003; Delhi, Los Angeles, and Washington D.C. for the year 2000). Detailed information of these 14 cities and metropolitan regions were given in Table 1. Per capita city emission statistics were reported by sector (electricity, heating & industrial fuels, industrial processes, ground transportation, waste, aviation and marine) for 10 cities (excluding Delhi, Tokyo, Manhattan and Washington D.C.) in literature [42] (see Additional file 1: Table S1 excluding waste, aviation and marine). For Delhi, Tokyo and Washington D.C., some sectors (electricity, heating and industrial fuels, and ground transportation) were reported as the energy sector along with other sectors (industrial processes, aviation, marine, agriculture, forestry, other land-use and waste) in literature [17] (see Additional file 1: Table S1 excluding agriculture, forestry, other land-use, aviation, marine, and waste). We added Manhattan, the central borough of New York City, as an additional example of small city area despite of no direct data, where we assumed its per capita sector emissions were the same as the New York City.

**Table 1:** Information of 14 Cities and metropolitan regions in this study

City or metropolitan region	Definition	Population (Kennedy)	Year (Kennedy)	Area (km^2^)	Total in-boundary FFE (MtCO_2_)	In-boundary FFE (tCO_2_/cap)
Bangkok	Bangkok Metropolis	5,658,953	2005	1574	27.53	4.86
Beijing	Beijing Municipality	15,810,000	2006	16,424	115.19	7.29
Shanghai	Shanghai Municipality	18,150,000	2006	6905	179.91	9.91
Delhi	Metropolis	13,200,000	2000	1508	12.72	0.96
Cape Town	City of Cape Town Metropolitan Municipality	3,497,097	2005	2451	6.12	1.75
Sao Paulo	Municipality	11,300,000	2011	1531^a^	10.58	0.94
Tokyo	Tokyo Metropolis	12,677,921	2006	1805	39.94	3.15
Greater Paris	Ile de France	11,532,398	2005	12,058	50.30	4.36
Greater London	Greater London	7,364,100	2003	1604	32.36	4.39
Los Angeles	County	9,519,338	2000	10,612	77.64	8.16
Manhattan	Borough	1,570,274	2005	69	7.44	4.74
New York City	City	8,170,000	2005	807	43.13	5.28
Washington D.C.	District of Columbia	571,723	2000	177.5	6.81	11.91
Greater Toronto	Greater Toronto	5,555,912	2005	7636	44.35	7.98

The population data for Delhi, Tokyo and Washington D.C. were taken from Kennedy et al. [17], for Manhattan was adopt from NYC Open Data [58], the population data for other 10 cities were used from Kennedy et al. [42]. Area information was based on GADM database except City of Cape Town and Washington D.C. The official area boundary from local governmental open source online for Cape Town [46] and Washington D.C. [47] were used in this study directly

^a^Area boundary for Sao Paulo referred to Ferreira MJ [59]

The sector inventory city emissions (primarily electricity, heating & industrial fuels, industrial processes, ground transportation) reported in literature were determined by multiplying activity levels data (e.g., fossil fuel combustion quantity) by appropriate emissions factors (e.g., emissions per fossil fuel combustion quantity). Here emission factors were based on the revised Intergovernmental Panel on Climate Change (IPCC) defaults or corresponding national values for local city and the activity levels data were adopt from local data specifically (e.g., energy statistical yearbook for cities). The detailed calculations and data sources by sectors used in this study are shown in Additional file 1: Table S2. Due to its importance in understanding the uncertainties in the reported data, based on descriptions in related literature, below we provide a brief synthesis for the main sectors how the emissions are calculated in inventory estimates.

Emissions associated with electricity are calculated as the product of electricity consumption (GWh), electrical line losses factor (including line losses in transmission and distribution), and the emissions factors (tCO_2_e/GWh). The electricity consumption data are collected by final users, namely, from local aggregation of consumer bills for electricity (excluding the electricity consumption from combined heat and power (CHP) plants in the city boundary and electricity provided by waste combustion, which are conventionally included in heating fuels and waste sectors, respectively). The city-specific emission factors vary widely depending on the mix of local power plants. Note that inventory electricity emission estimates include emissions from power plants outside of city boundary.

Emissions from ground transportation primarily include gasoline and diesel, with natural gas and fuel oil consumptions of small amounts, but do not include emissions produced by electrified modes of transportation (e.g., subways) which is allocated in the electricity sector. Emissions (tCO_2_e) for ground transportation are computed to sum all emissions from fossil fuel consumptions by ground transportation sector, after multiplying energy contents (TJ) of fuels by respective emissions factors (tCO_2_e/TJ) from IPCC for each fuel type. The energy contents are calculated as the product of net calorific value (TJ/m^3^) and fossil fuel consumption (ML). Three different methods are employed to calculate the fossil fuel consumption: (1) local fuel sales data where available, this is preferred by the IPCC and appropriate where the number of daily commuter trips across the city boundary is smaller than the number of trips within the city; (2) vehicle kilometres travelled (VKT) (billion km, values derived from modeling or vehicle counting surveys, vary by city) divided by fuel efficiency for specific vehicles (km/L). This approach is appropriate for central cities where the number of cross-boundary commuter trips is large and the fuel sales may occur out of the central city boundary; (3) scale fossil fuel consumptions from state, provincial, or wider regional data based on the assumption that vehicles in the city travel the same average annual kilometres as in the wider region, if there is no reliable data in the city. The scaling factor may be determined by motor vehicle registrations or population corresponding closely to the total travel commute times.

Emissions in the category of heating and industrial fuels include fossil fuel consumption for heating demands (e.g., district heating, cooking, and water heating), fossil fuels consumed by CHP facilities within the urban region (primarily natural gas and oil), and combustion of fossil fuels in industry; Emissions from industrial processes are non-combustion emissions released during chemical processes (e.g., cement manufacturing and limestone consumption), which can be determined as a product of the product quantity (Mt) and the emission factor (kg CO_2_/t) of the process.

### ODIAC CO_2_ emission data

In order to estimate fossil fuel CO_2_ emissions in the boundary of each city or metropolis, we utilized the 2017 version of ODIAC CO_2_ emissions data which was available at the time of this study [4, 34–37]. This global monthly CO_2_ emission dataset spans from 2000 to 2017 and downscales the national level emission statistics from the Carbon Dioxide Information Analysis Center (CDIAC, version 2014) [28] and the global fuel statistical review data from the British Petroleum Company (after 2014) to 1 km resolution. The downscaling is based on 1 km globally nighttime light data from the Defense Meteorological Satellite Program (DMSP) satellite and worldwide large point emission sources (power plants/companies) from Carbon Monitoring for Action database (CARMA) [43]. CO_2_ emission at country level was first assigned at manually corrected geographical locations of large point sources according to their emission quantities reported in CARMA. Then the remaining non-point source emission (industrial, commercial and household consumption, land transportation) was distributed using nighttime light intensity as surrogate, assuming that nighttime light intensity correlates with CO_2_ emissions directly and the correlation was uniform and linear across countries worldwide. Note that although the emissions from cement production should be regarded as point sources, these parts of emissions were distributed as nonpoint source emissions in ODIAC due to the lack of data [34]. This might show up as a small difference between ODIAC and emissions reported by cities.

### City boundaries data

In order to have a consistent comparison, we identified the city boundaries to be as close to the boundaries used in inventory estimates in literature as possible. Since the exact city boundaries used in such literature are often not clear, we relied on listed city name and area metadata for helping to identify consistent city boundaries. We started from the database of Global Administrative Areas (GADM) version 2.0 [44] to define most cities’ boundary excluding Cape Town (City of Cape Town Metropolitan Municipality) and Washington D.C. This database features high-resolution vector-form boundary data, coming with three main levels for most regions: country level (L0), state/province level (L1), and county/city level (L2). The extracted city areas from GADM were then compared with corresponding city area information listed in inventory estimates literature [16, 45]. We found that the city areas differed on average only by 2.3%, with largest difference of 8.2% for Shanghai (see Additional file 1: Table S3). For Cape Town and Washington D.C., in order to have boundaries close to reported city area in inventory literature (Additional file 1: Table S3), we used instead the boundaries from Municipal Demarcation Board [46] and Washington D.C. government open datasets [47] separately.

**Fig. 2:**
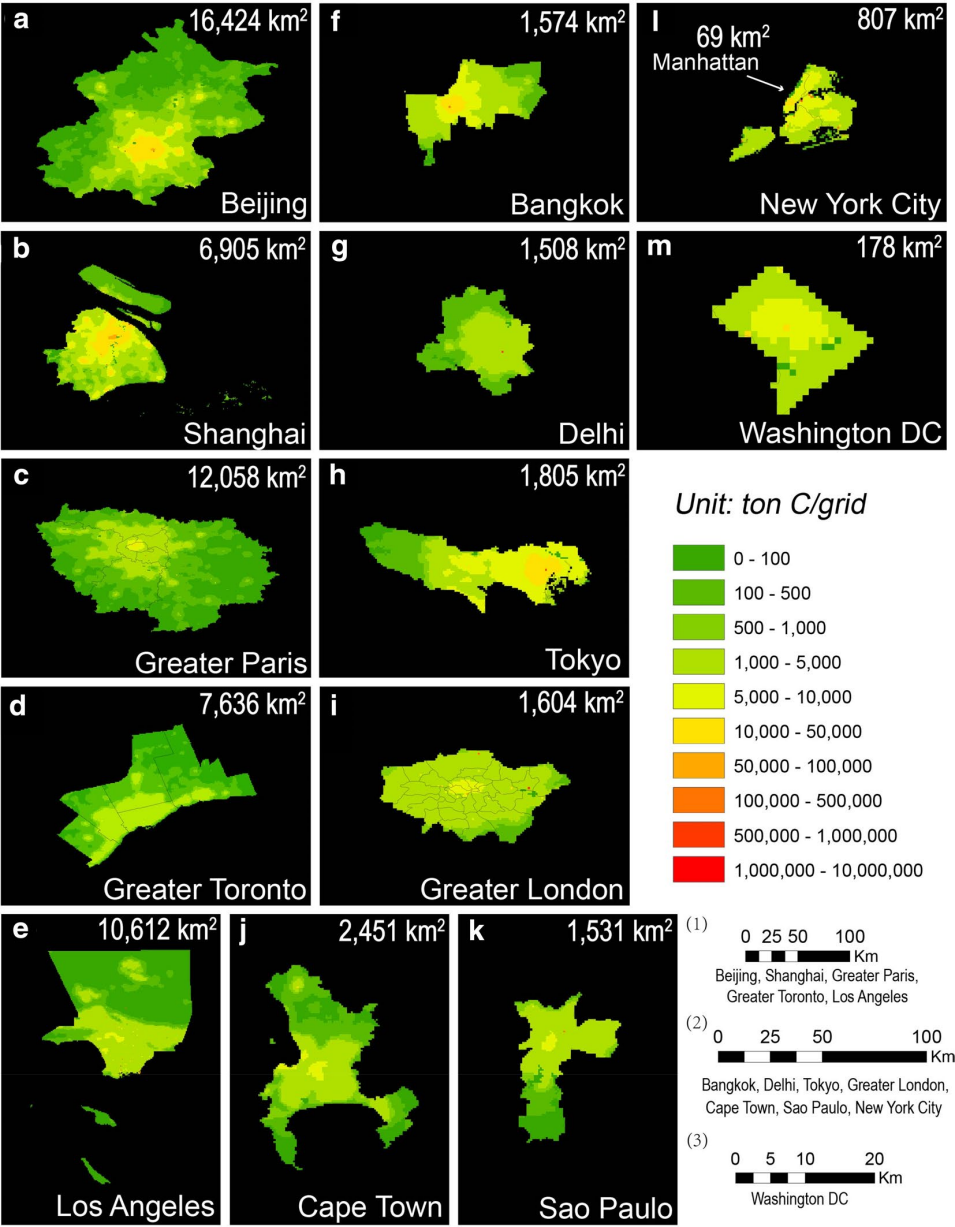
Spatial distributions of fossil fuel emissions (tC/grid) for 14 cities and metropolitan regions in the year listed in Table 1 based on the ODIAC inventory (1 km resolution). Manhattan is located in the upper left part in New York City showed in region (i). Grid points with high emissions (shown red) clearly represent some large point sources. Three scale bars were chosen in this distribution. Scale bar (1) corresponds to region (**a**–**e**), scale bar (2) to region (**f**) to (l) and scale bar (3) to Washington D.C.

After identifying the boundaries for the 14 city and metropolis areas, we computed the cities’ total CO_2_ emission by overlaying with both total emission and point source emission data from ODIAC, for years corresponding to those reported in inventory studies. The spatial patterns of ODIAC city emissions and the city boundaries were presented in Fig. 2 (ODIAC data with years corresponding to local inventory estimates, as listed in Table 1, were used in this figure), and per capita large point source emissions in each city were listed in Additional file 1: Table S1.

### Calculation of in-boundary CO_2_ emission

For inventory estimates, we first determined which sector or part of sector contributes to cities’ total in-boundary CO_2_ emissions. For most sectors, although the sector emissions were reported as GHG emission instead of CO_2_ emissions, we used the data as CO_2_ emissions by neglecting (except for the waste sector) non-CO_2_ gases emitted from the sectors (for simplicity, we refer to fossil fuel CO_2_ emissions as emissions in most cases except for waste). Sectors of heating and industrial fuels, industrial processes are more straightforward and could generally be considered to occur within city boundaries, therefore we directly used their numbers as reported from literature. A major part of emissions from ground transportation likely occurs in large city areas, and because better alternative estimates do not exist at global scale, we also treated reported emissions from ground transportation as in-boundary emission. For the electricity sector, reported electricity consumption statistics normally include a significant part of electricity generated outside of the consumer city. For example, 71% of total electricity in Beijing was provided from power plants out of its boundary in 2006 [48]. Therefore, we adopt an alternative way to estimate in-boundary city electricity emissions. For the aviation and marine transportation sectors, the city statistics were calculated in terms of full volumes of fuels loaded at the airports or harbors of the city. However, most fuels were consumed and emitted as GHG outside of city boundaries; only very limited emissions were produced from take-offs and landings within the city boundaries, therefore we excluded these sectors from in-boundary city emissions. As for emissions from waste, since most is methane as a result of organic waste decomposing at low oxygen, waste emission was also excluded in counting in-boundary city CO_2_ emissions.

For in-boundary emission from the electricity sector, instead of using reported inventory data, we relied on the CARMA large point sources (power plant) emission database, which was manually corrected for location errors and provided as an annual point source emission layer from ODIAC. By overlaying cities’ boundaries with the point source emission layer, we computed electricity in-boundary emission in the same year as the inventory sector emission statistics for each city. For example, for Beijing in 2006, we found the in-boundary emission contributes to 29% of reported emission from Beijing’s electricity sector, consistent with previous literature [48]. Then we added the in-boundary large point source emission with emissions from heating and industrial fuels, industrial processes and ground transportation as reported from literature to determine the inventory in-boundary emission for each city. Note that our comparison between ODIAC and inventory estimates do not include differences in the electricity sector as the same point source dataset was used in both estimates.

For calculation of emissions from ground transportation, the method based on fuel sales data was applied to Bangkok, Cape Town, Beijing, Shanghai, Greater Toronto, and Sao Paulo; the VKT method was established for Greater London, and New York City; and the scaling approach was used for Los Angeles [23]. The method choices of fuel consumption calculation for ground transportation in other cities are unknown to the authors. Data for emissions from industrial processes is only available for eight cities: Beijing, Shanghai, Sao Paulo, Tokyo, Greater Paris, Los Angeles, Washington D.C., and Greater Toronto. For Bangkok, Delhi, Cape Town, Greater London, and New York City, there are no recorded emissions. The magnitude of emissions in this category is usually small, but emissions in specific cities are quite substantial. For example, emissions from industrial facilities in Greater Toronto are larger than 100,000 t CO_2_e reported by Environment Canada and could not be neglected [23].

For most of the cities studied, all sector emission data are available in literature, and we assigned zero value to “unknown” or “negligible” in the columns named industrial processes and marine in the tables. For Delhi, Tokyo and Washington D.C., we proxied the fractions of electricity sector emission from their reported emission for the total energy sector (electricity, heating and industrial fuels and ground transportation), by referring to cities with similar geographical location and social economic development level. In general, larger emission proportion from electricity associates with less clean energy sources used to generate electricity in cities. For Delhi where coal is the main fuel supply for electricity generation [49], we chose the value of 45% for proportion of electricity in energy. This was slightly higher than some other big cities in the Asian region, such as Beijing and Shanghai, which were also densely-populated megacities plagued by poor air quality. For Washington D.C., we assumed 35% as the proportion, a value in between emission percentages for the other two U.S. cities studied (New York City and Los Angeles). Similarly, for Tokyo, we assume 36% of emissions from energy contributed to electricity sector emission. This value was chosen to be roughly the average of some megacities in developed countries such as New York City, Los Angeles and Greater London. These values should be treated with caution as actual proportion values may easily differ from our partially subjective assumptions by 10% or more, so results from Delhi, Tokyo, Manhattan and Washington D.C. might not be as reliable as the other ten cities. Nevertheless, sensitivity analyses show our main results of comparing the two emission estimates remain largely the same with different proportion assumptions (see Additional file 1: Table S4).

After the emissions of electricity division from total energy in Delhi, Washington D.C., Manhattan and Tokyo, like the other cities we added emission from large point sources to emission from remainder of energy consumption and industrial processes to obtain per capita in-boundary CO_2_ emissions for these cities. Then for the total in-boundary emission in the 14 cities and regions, we multiplied the reported population data in the same year reported in literature [17, 42] with the per capita CO_2_ emissions in each city/region to compare with ODIAC-based in-boundary city emission estimates.

## Results

### Inventory scope 2 and in-boundary city emissions

**Fig. 3:**
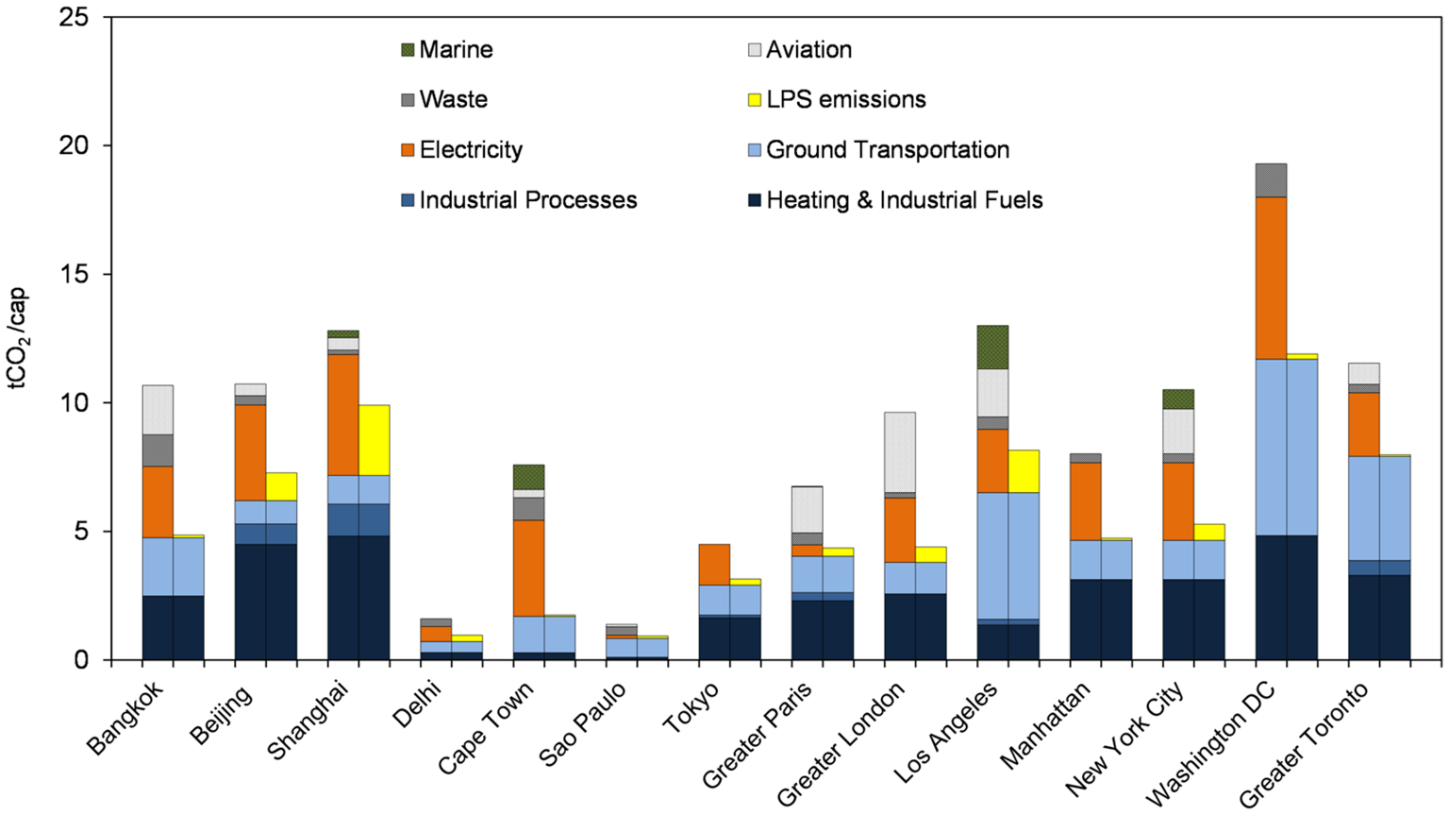
Emissions per capita (tCO_2_/cap) for the 14 cities and regions worldwide. Left bar: emission statistics in Scope 2 adapted from the prior literature [17, 42], composed of emissions from heating and industrial fuels, industrial processes, ground transportation, electricity, waste, aviation and marine; Right bar: in-boundary (Scope 1) emissions for the same cities and regions, where in-boundary large point source emission totals replaced emission from the electricity sector

Per capita emissions for these 14 cities and regions are shown in Fig. 3. The scope 2 emissions (left bar) display a wide range from 1.39 to 19.3 tCO_2_ (tCO_2_ equivalent for waste) per capita. The U.S. capital has the highest per capita emissions by a wide margin, followed by Los Angeles and Shanghai, while Sao Paulo has the lowest emissions thanks to its reliant on renewable energy sources such as hydropower and biogas. The high per capita emissions in Washington D.C. come from a large contribution of ground transportation, heating and industrial fuels, electricity consumption and (to a lesser extent) waste. It is also the top emitter among these cities and regions with in-boundary emissions of 11.9 tCO_2_/cap, with the next highest city being Shanghai (9.9 tCO_2_/cap) followed by Los Angeles at 8.2 tCO_2_/cap. The order reversal of Los Angeles and Shanghai is related to the larger emissions from international marine and aviation in Los Angeles.

There are a couple of interesting observations from the sector specific emissions (see Fig. 3 and Additional file 1: Table S1). Per capita ground transportation emissions are generally high in Washington D.C., Los Angeles and greater Toronto, all North America cities where commuting with cars is probably more attractive than public transportation due to high car ownership, relatively low population density and/or people live further away from their workplaces. Emissions from industrial processes are generally small in cities: the highest number is found in Shanghai (1.25 t CO_2_/cap), a city known for its strong industry presence with large production of steel, cars etc.; Beijing and Greater Toronto are the other cities that exceed 0.5 t CO_2_/cap emission for industrial processes. For the electricity sector, comparing emission from large point sources within city boundaries with inventory statistics show interesting differences in cities’ reliance on external regions to provide power. While Washington D.C. has the largest CO_2_ emissions from electricity sector (6.3 t CO_2_/cap), most of the electricity consumed is not generated by large power plants in the city boundary (only 0.21 t CO_2_/cap). Bangkok, Manhattan, Cape Town and greater Toronto also rely on out-of-boundary or non-large point sources to meet most (over 90%) electricity consumption demands. In contrast, partially thanks to its strong industry, Shanghai has the highest in-boundary large point sources emissions (2.73 t CO_2_/cap, 59% of its electricity consumption), near 65% higher than the next city Los Angeles (1.65 t CO_2_/cap, 67% of its electricity consumption), and a factor of 54 more than the lowest city of this category (Cape Town, 0.05 t CO_2_/cap). The largest CO_2_ emissions per capita from aviation in Greater London and from marine in Los Angeles reflect the roles of Greater London and Los Angeles as international gateway centers. The high aviation emission in Bangkok is probably related to its abundant tourism.

### Comparing ODIAC emissions to local inventory estimates

**Fig. 4:**
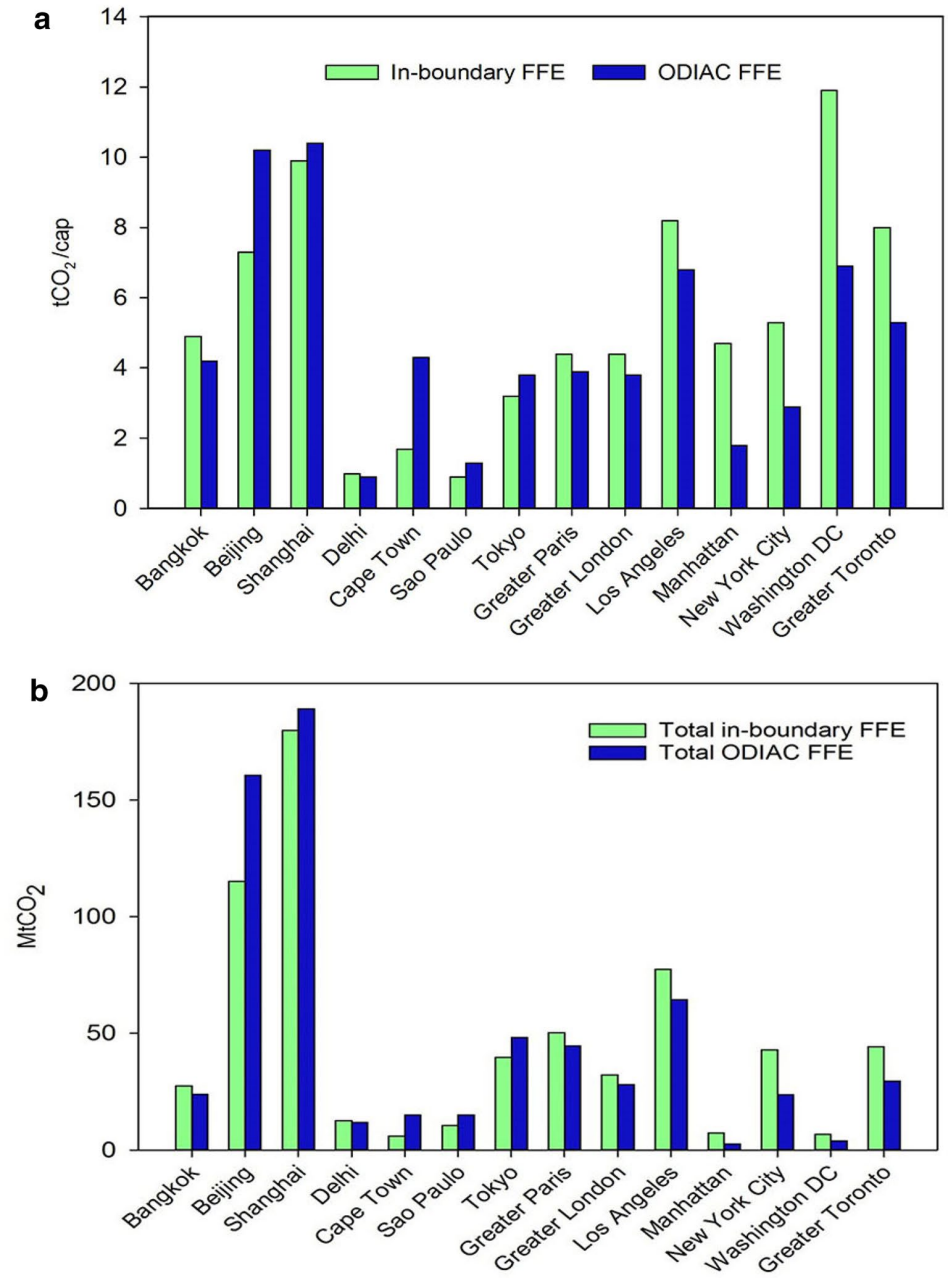
**a** Per capita emissions for the 14 cities and metropolitan regions, based on in-boundary inventory (green) fossil fuel emissions (FFE) and ODIAC dataset (blue) (tCO_2_/cap). **b** Total CO_2_ emissions for cities and metropolitan regions based on in-boundary fossil fuel emission (green) and ODIAC dataset (blue) (MtCO_2_)

Comparison of per capita and total CO_2_ emissions based on ODIAC and in-boundary CO_2_ emissions are illustrated in Fig. 4a, b, respectively. Despite distinct approaches in city emission estimates, the total and per capita emissions in half of the cities and regions are remarkably similar, with less than 30% difference (Bangkok, Shanghai, Delhi, Tokyo, Greater Paris, Greater London, and Los Angeles). The ODIAC-based city emissions range from 0.9 t CO_2_/cap in Delhi to 10.4 t CO_2_/cap in Shanghai. Meanwhile, in-boundary CO_2_ emissions per capita in Delhi and in Shanghai are 1.0 t CO_2_/cap and 9.9 t CO_2_/cap, respectively, which are very close to ODIAC emissions with less than 10% difference. The CO_2_ emissions per capita based on ODIAC is notably higher than in-boundary CO_2_ emissions in Cape Town, Sao Paulo, and Beijing, with a difference of 148%, 43% and 40%, respectively. On the other hand, the ODIAC dataset exhibits lower estimates in North American cities and regions: Manhattan (− 62%), New York City (− 45%), Washington D.C. (− 42%), and Greater Toronto (− 33%).

The ODIAC nighttime light-based emission disaggregation is known to work less well in developing countries compared to developed countries, as the nightlight intensity sometime correlates poorly with human activity in developing countries [50, 51]. In general, lots of low emissions in non-urban areas are missed in the light-based emission disaggregation for developing countries, and they tend to be assigned to cities instead [34]. The manufacturing industry tends to have relatively high emission, and the industrial fossil fuel combustion and industrial processes may take place outside big cities. Factories in developing countries may also not be so well lit as in developed countries, especially compared to their relatively high emission output. This may partly explain the overestimation of ODIAC-based city CO_2_ emissions in the central metropolis cities of developing countries such as Beijing, Cape Town and Sao Paulo. This is especially the case for Cape Town (the city with largest discrepancy), the well-lit tourist and high-tech hub of South Africa, where few power plants (see Table 1) or factories are located. However, there does not seem to have a consistent pattern as the two estimates agree reasonably well in some other cities in developing countries such as Bangkok, Delhi and Shanghai. In those cases, a closer examination is needed. Shanghai for example has a strong industry within the city border, which may lead to error-offsetting that ends up producing a similar number as ODIAC-based estimate. And most power plants and vehicles agglomeration within Delhi can contribute to error-offsetting.

On the other hand, ODIAC-based emission may underestimate the emissions in North American cities/regions, especially in cold regions, as this nightlight-based approach might underestimate emission from heating: more heating consumption is needed in cities with cold winters, while the cities’ lights are not necessarily brighter than southern cities where less heating is needed. Therefore, disaggregating non-point source emission from nighttime light alone in a large country with quite varied climate condition might lead to overestimating emission in warmer cities and underestimating emissions in colder cities, while ODIAC does include emission seasonality. Note that although the northern cities may require less cooling in summer which potentially offset the emission from increased heating in winter, cooling consumes electricity which is not disaggregated based on nighttime light in ODIAC. Additionally, in the case of Washington D.C. (to a lesser extent also Manhattan, New York City, and Greater Toronto), it is likely that in-boundary emissions might be overestimated because of the way ground transportation emission is calculated. In cities like Washington D.C. and New York City, vehicles registered there may travel not only in the limited city area but also in nearby states as the in-city travel distance is short in general, therefore only part of the reported CO_2_ emissions from ground transportation may occur in the city. This effect is much weaker in larger city areas such as Los Angeles.

The total CO_2_ emissions based on ODIAC have a range of 2.8 MtCO_2_ in Manhattan to 189.1 MtCO_2_ in Shanghai, while the total in-boundary emissions range from 6.1 MtCO_2_ in Cape Town to 179.9 MtCO_2_ in Shanghai (Fig. 4b). Total city emissions in Beijing and Shanghai stand out, as they are cities with largest total populations, even though their per capita emissions are comparable with some other cities in developed countries. The total emissions comparatively go down in Cape Town and Washington D.C., due to their relatively small population numbers.

## Discussions

To our knowledge, the in-boundary emission estimates and comparison with ODIAC for 14 large cities and metropolis areas as presented in the study is one of the first at this scale, and the differences at city scale suggest some levels of confidence in using ODIAC dataset as a first prior for city emissions. We note that although a possible error under 30% or 10% seem fine given the relatively simple spatial disaggregation method employed in ODIAC, city emissions based on ODIAC may only track the long-term progress instead of year-to-year variations of emission related to emission reduction policies. In the data-rich U.S. recently there has been increasing effort to quantify emission at local scale. Gurney et al. [22] compared ODIAC and the Hestia dataset in four American urban areas, and they found an even smaller whole-city difference (− 1.5%) for Los Angeles Basin. However, Gurney et al. [21] reported that Hestia emissions are 10.7% larger than the local emission estimates in Los Angeles. Additionally, both the study area (17,795 km^2^ versus 10,612 km^2^ in our study) and year (2011 vs 2000 in our study) are different from our study. The whole-city difference in other cities were also found to be small and within the range of our estimates, up to + 20.8% for Salt Lake City. Although confined to four U.S. cities due to lengthy development time requirements, the high spatiotemporal details of the Hestia dataset allows for grid level analyses and spatial correlation computation, which shows much larger differences at 1 km grid level, with the median difference ranged from 47 to 84% (largest discrepancies were dominated by the on-road sector and large point sources), and moderate spatial correlations between 0.34 and 0.68. Oda et al. [4] implemented a similar comparison using ODIAC and a national-level multi-resolution inventory over the domain of Poland. The study showed ~ 40% pixel level differences over the cities. New sub-city bottom-up inventory estimates similar to Hestia, especially in megacities from developing countries, would provide much needed insights on the error estimations for local emission inventories.

In this study, we have so far focused on the potential downscaling biases and errors of the ODIAC dataset when used on city scale, in order to understand its difference with local inventory-based estimates. It is worth noting that many fundamental difficulties exist when using ODIAC, which was not originally designed for urban CO_2_ emission monitoring purposes [4, 22]. Also, the nighttime light data ODIAC use also has some minor location errors/shifts that may affect in-boundary emission estimates at city scale, especially for point sources at the border of cities. Nevertheless, we observed some general patterns in the differences which, when combined with known shortcomings of ODIAC data, could be plausibly explained. However, we acknowledge that our assumptions to derive in-boundary city emissions based on reported sector emission data could potentially render the inventory-based city emissions equally uncertain, therefore the differences in these two emission estimates (especially for the few cities with aggregated sector emission data) need to be interpreted with care.

Uncertainty estimation for inventory CO_2_ emissions at city level is challenging as the data are only available from a few sources, for which the uncertainty estimations are rarely reported, and usually rely on “expert judgment”. Nevertheless, it is critical and necessary to evaluate the uncertainties of emissions inventories especially at local and city scales. While it is beyond our capacity to quantify uncertainty of inventory estimates for every sectors in each city, in the following part of this section, we attempt to quantify some uncertainties for fossil fuel CO_2_ inventories at city scale, by comparing in-boundary inventory emission estimates used in this study with some other sector inventory emission products in the United States. Specifically, we focus on ground transportation and electricity sectors. We also discuss the important issue of inventory data versioning and other sources of uncertainties.

### Uncertainty associated with emissions from ground transportation

Our assumption that inventory ground transportation emission is in-boundary emission may cause significant errors especially in smaller cities, as a considerable portion of vehicles refuel or travel can occur outside city boundaries, which is not easily differentiable from local activity data. In order to evaluate the uncertainty from ground transportation emissions, we compared inventory city emissions from ground transportation sector with emissions reported in the database of Road Transportation Emissions (DARTE) in the same year for the four U.S. cities (Washington D.C. and Los Angeles in 2000, and New York City and Manhattan in 2005). DARTE is an emission inventory which can estimate CO_2_ emissions from US road transportation with an annual 1 km resolution for 1980–2012, based on archived roadway-level vehicle traffic data [52, 53], therefore it could potentially provide more accurate in-boundary emission from ground transportation for U.S. cities. The inventory data shows higher ground transportation CO_2_ emissions consistently in all of these four American cities and regions, relative to DARTE (see Additional file 1: Table S8). The relative differences in New York City and Los Angeles, computed by (inventory data-DARTE)/DARTE *100%, are 7.9% and 9.3%, respectively, while the relative differences are more than 100% in Washington D.C. and Manhattan. The results imply that uncertainty of CO_2_ emissions estimation by ground transportation sector at larger city or region level can be much less than smaller city or county.

### Uncertainty associated with emissions from electricity sector

While the city emission differences in our comparison do not directly come from the electricity sector (as both in-boundary estimates and estimates from ODIAC use the same large point source emission layer), it is important to discuss whether the CARMA data is sufficient for estimating in-boundary emission from electricity. To evaluate this uncertainty source, we compared power plant emissions from the CARMA (Carbon Monitoring for Action) database adopted in ODIAC and the eGRID (the Emissions & Generation Resource Integrate Database) [54] published by the United States Environmental Protection Agency for four American cities and regions (New York City, Washington D.C., Los Angeles, and Manhattan). The eGRID database is an inventory of electric power systems, contains emission data of all electric power sectors and power plant’s physical location information in the United States, and can provide aggregated data by total, state, county and electric grid boundaries. We used emissions generated by individual power plant from eGRID database for the year 2000 and 2005 to compared with emissions from electricity based on CARMA/ODIAC data for Washington D.C. and Los Angeles in 2000, and New York City and Manhattan in 2005. The relative differences calculated as (CARMA-eGRID)/eGRID *100% are shown in Additional file 1: Table S9. Emission in each city or county from CARMA is consistently lower than from eGRID. The largest relative difference is − 93.5% in Manhattan, while the least relative difference is − 7.2% in Los Angeles which features the largest area of the four. Emissions from CARMA are lower by 66.6% in New York City and by 37.2% in Washington D.C. relative to the eGRID. The imprecise plant-specific location in CARMA may account for these differences. Wheeler and Ummel [43] also indicate that CARMA in some cases underestimate total emissions for smaller geographic regions because relevant geographic information is sometimes unavailable, Therefore, the in-boundary emission estimates for the electricity sector based on CARMA data may underestimate the actual in-boundary emission especially for small regions.

As we empirically estimated the fractions of emission from electricity sector for Delhi, Tokyo and Washington D.C. in absence of detailed data, for these three cities the fractional estimations also contribute to sources of uncertainties in inventory in-boundary emissions. While we cannot provide an absolute uncertainty range as the three cities could potentially deviate outside the range based on similar cities, sensitivity analyses were performed on the empirical fraction assumptions. By applying the maximum/minimum electricity portion assumptions according to similar cities, the difference between ODIAC and local inventory city in-boundary CO_2_ emission estimates are 7–16% for Delhi, 5–28% for Tokyo, and 38–49% for Washington D.C. (see Additional file 1: Table S4).

### Uncertainty associated with data versioning

In inventory emission reporting, it is common to update previous emission estimates, however, the data versioning is often poorly documented; emission data for some sectors could be collected in prior data year, and there could also be errors in previous data versions [4]. While it would be quite difficult to track down data versioning for the inventory emission sources used in this study (the differences caused by an offset of 1 or 2 years in some sectors will also likely not be substantial relative to the city-scale emission difference between inventory and ODIAC estimates), we believe a better reporting with clear data versioning is urgently needed for transparent emission accounting in inventory compilation efforts. Data errors can be large in some cases, for example, Kennedy et al. [55] reported erratum about fuel consumption data for the city of Cape Town in 2005. Some of the fuel data in the previously paper was erroneous due to the relative data refers to national sales data from local refinery in the city of Cape Town, not required fuel consumption data in the city. The revised emissions are reported as 7.6 t CO_2_ e/cap in Cape Town, much lower than the previous result of 11.6 t CO_2_ e/cap emissions by 34.5%. We adopted the revised data in our study, however, we foresee that such errors may recur in the future and could potentially greatly undermine emission reduction efforts from the cities; it is also potentially exploitable as some emission reduction may simply come from updating to a later data version. Therefore, we recommend systematic versioning of inventory emission data as a common practice to promote transparent city emission monitoring in the future.

### Other uncertainty sources

In addition to the above uncertainties, there are uncertainties associated with some other sources. Firstly, only a few government sources about emission are reported, and without reported estimates of uncertainties. In addition, no other data sets are available with these unknown uncertainties. For example, emissions by industrial processes such as from the brick and clay plants in Cape Town are unknown. It’s uncertain whether the combustion emissions during flaring are included and no other available data to estimate this uncertainty. Secondly, large uncertainty may exist for IPCC default emission factors, which will contribute to the uncertainties in this study. Liu et al. [56] found that the emission factors for coal in China are average 40% lower than the IPCC default emission factors. Shan et al. [57] also found that the aggregated CO_2_ emissions in China calculated by updated emission factors and the apparent energy consumption are 12.69% lower than emissions calculated by IPCC default emission factors and the traditional approach. Multiple different emission estimation methods, accompanied by transparent documentation and data versioning, would be fundamental in quantifying data uncertainties and making emission reduction goals trackable.

## Conclusions

In this study we have summarized bottom-up in-boundary city CO_2_ emissions estimates based on large point emission sources and inventory statistics for 14 large cities and metropolis areas across the globe. These city CO_2_ emission statistics are compared with downscaled city emissions based on the ODIAC dataset. Results show that despite of mostly relying on satellite nighttime light data for spatial disaggregation of emissions, the ODIAC dataset agree with inventory-based in-boundary CO_2_ emission reasonably well (difference within 30%) for half of the cities examined. The relatively good agreement, in conjunction with ODIAC’s high spatial resolution (1 km) and timely update of its monthly global database, suggest that the ODIAC dataset has promising potential as a good first source for prior estimate of city-level CO_2_ emission, especially as the in-boundary city emission estimates, while urgently needed, are not easily available in all cities. The total and point source emission information from ODIAC could be useful for inversion modeling and comparing with satellite CO_2_ observations.

However, there are still many uncertainties at city scale in both the inventory in-boundary city emission estimates and ODIAC-based estimates. While we attempted to compile in-boundary estimates for 14 large cities and metropolis areas, more cities especially in the developing countries are needed in the comparison to further improve our understanding on the uncertainties. Much effort is needed in the future towards establishing an accurate inventory in-boundary city carbon emission dataset with good global coverage, which would be highly valuable for evaluating the effectiveness of various emission reduction measures pledged at city scale. On the other hand, the 1 km ODIAC dataset will continue to evolve and improve on the potential shortcomings in the process of being applied in various carbon emission monitoring and modeling efforts and compared to other high-quality dataset at local scale. Through our preliminary comparison effort, it is our hope to draw more attention from the community to city-scale in-boundary emissions calculation and more engagement with policy makers, which can add crucial contribution to accountable local climate mitigation policies in the future.

## Supplementary information

**Additional file 1.** Additional tables.

## Data Availability

The data supporting this research are available listed in the paper and the references. Additional data are available upon request to corresponding author.

## Abbreviations

EIs: Emission inventories
GHG: Greenhouse gas
OCO-2: Orbiting Carbon Observatory 2
GOSAT: Greenhouse Gases Observing Satellite
TanSat: Chinese Carbon Dioxide Observation Satellite Mission
OCO-3: Orbiting Carbon Observatory 3
GeoCarbon: Operational Global Carbon Observing System
EDGAR: Emission Database For Global Atmospheric Research
CDIAC: Carbon Dioxide Information Analysis Center
FFDAS: Fossil fuel data assimilation system
ODIAC: Open-data inventory for anthropogenic carbon dioxide
IPCC: Intergovernmental panel on climate change
CHP: Combined heat and power
VKT: Vehicle kilometres travelled
DMSP: Defense meteorological satellite program
CARMA: Carbon monitoring for action
GADM: Global administrative areas
L0: Country level
L1: State/province level
L2: County/city level
DARTE: Database of road transportation emissions
eGRID: Emissions and generation resource integrated database
FFE: Fossil fuel emissions
