# Supplementary material for: Comparing a global high-resolution downscaled fossil fuel CO2 emission dataset to local inventory-based estimates over 14 global cities
Source: Carbon Balance Manag. 2020 May 19;15:9. doi: 10.1186/s13021-020-00146-3 (PMC7238606; doi:10.1186/s13021-020-00146-3)
Supplement: Supplementary file 1 — Additional file 1. Additional tables. [file 13021_2020_146_MOESM1_ESM.pdf]

Supplementary Information

Contents

Table S1. Per capita CO_2 emissions by sector for cities and metropolitan regions

Table S2. Detailed calculations for the inventory-based urban emissions and data sources by sectors

Table S3. Comparison of area for cities and metropolitan regions

Table S4. Fractional estimation of emission from electricity sector

Table S5. New York City population by Manhattan

Table S6. In-boundary fossil fuel emissions for cities and metropolitan regions

Table S7. Differences between calculations of emissions from ground transportation

Table S8. Differences between CO_2 emissions by transportation sector from DARTE and inventory -based data

Table S9. Differences between CO_2 emissions by electricity sector from eGRID and inventory -based data

Table S1. Per capita CO_2 emissions by sector for cities and metropolitan regions

City	Electricity (tCO_2e/cap)	Heating & Industrial Fuels (tCO_2e/cap)	Industrial Processes (tCO_2e/cap)	Ground Transportation (tCO_2e/cap)	Energy (tCO_2e/cap)	Electricity/ Energy (%)	Heating & Industrial Fuels/ Energy (%)	Ground Transportation/Energy (%)	Large Point Source (tCO_2/cap)
Bangkok	2.77	2.49	0	2.27	7.53	36.8	33.1	30.1	0.10
Beijing	3.72	4.50	0.79	0.91	9.13	40.7	49.3	10.0	1.09
Shanghai	4.71	4.82	1.25	1.11	10.64	44.3	45.3	10.4	2.73
Delhi	0.59	0.30	0	0.42	1.3	45.0	23.0	32.0	0.25
Cape Town	3.74	0.29	0	1.41	5.44	68.8	5.3	25.9	0.05
Sao Paulo	0.12	0.11	0	0.74	0.97	12.4	11.3	76.3	0.09
Tokyo	1.58	1.65	0.1	1.17	4.4	36.0	37.4	26.6	0.23
Greater Paris	0.44	2.31	0.32	1.41	4.16	10.6	55.5	33.9	0.32
Greater London	2.5	2.58	0	1.22	6.3	39.7	41.0	19.4	0.59
Los Angeles	2.46	1.37	0.22	4.92	8.75	28.1	15.7	56.2	1.65
Manhattan	3.01	3.13	0	1.53	7.67	39.2	40.8	19.9	0.08
New York City	3.01	3.13	0	1.53	7.67	39.2	40.8	19.9	0.62
Washington D.C.	6.3	4.84	0	6.86	18	35.0	26.9	38.1	0.21

Greater Toronto	2.47	3.30	0.57	4.05	9.82	25.2	33.6	41.2	0.06

For Washington D.C., Manhattan, Delhi and Tokyo, the CO_2 emissions by sector can be used from this table. Due to data limitation, we needed to divide the heating & industrial fuels and ground transportation from energy after the allocation of electricity proportion for these four regions. Here emissions from energy in Table S3 include emissions from stationary, mobile combustion and fugitive sources (specifically from industrial fuels, electricity & heat production, and ground transportation excluding aviation and marine). Referring to the literature [1], we found that more than 30% emissions can be attributed to ground transportation in some North American cities. And based on the similar fuel economy and habits of vehicle usage in America, we assumed that the average ground transportation proportion of New York City and Los Angeles can be used as the proportion of ground transportation for Washington D.C.. For Manhattan we also assumed the same ground transportation proportion as New York City. For Delhi we adopted the proportion of ground transportation from the literature [2] directly. According to social economic development, population density and quality of public transit, we assumed the average ground transportation proportion for Tokyo can be empirically established on European cities. So the average ground transportation proportion of Greater London and Greater Paris was employed for Tokyo. After the proportion division of ground transportation and electricity from energy, the remainder of energy would be the proportion of heating & industrial fuels.

Table S2. Detailed calculations for the inventory-based urban emissions and data sources by sectors

City	Source	Energy	Electricity	Heating & Industrial Fuels	Ground Transportation(Local Fuel Sales)	Ground Transportation (VKT)	Ground Transportation (Scaling)	Industrial Processes
Bangkok	Kennedy et al.(2010),Phdungsilp (2006)		√	√	√*	√		Unknown
Beijing	Sugar et al.(2012)		√	√	√*			√
Shanghai	Sugar et al.(2012)		√	√	√*			√
Delhi	Mitra et al.(2003)	√	?	?	?	?	?	?
Cape Town	Kennedy et al.(2011)		√	√	√*			Unknown
Sao Paulo	Hoornweg et al.(2012)		√	√	√*			√
Tokyo	Tokyo Metropolitan Government, 2009	√	?	?	?	?	?	√
Greater Paris	ARENE/ADEME (2010)		√	√	?	?	?	√
Greater London	Kennedy et al.(2010)		√	√		√*		Unknown
Los Angeles	Kennedy et al.(2010)		√	√			√*	√
New York City	Kennedy et al.(2010)		√	√		√*	√	Unknown
Washington D.C.	DC Dept. Health (2005)	√	?	?	?	?	?	?
Greater Toronto	Kennedy et al.(2010)		√	√	√*		√	√

√ data is available   *final method choice of emission from ground transportation

?data and method are unsure or unknown (to the authors)

Table S3. Comparison of area for cities and metropolitan regions

City	total land Area (km^2_Kennedy_2009)	total land Area (km^2_Kennedy_2016)	Area (GADM) km^2	Area differences (%)
Bangkok	1,569	N/A	1,574	0.31
Beijing	N/A	16,411	16,424	0.08
Delhi	N/A	1,483	1,508	1.64
Greater London	1,579	N/A	1,604	1.58
Los Angeles	10,518	N/A	10,612	0.89
Manhattan	N/A	N/A	69	N/A
New York City	789	N/A	807	2.22
Greater Paris	N/A	12,011	12,058	0.39
Shanghai	N/A	6,341	6,905	8.17
Tokyo	N/A	N/A	1,805	N/A
Greater Toronto	7,195	N/A	7,636	5.77
Washington D.C.	177*	N/A	166	6.04
Sao Paulo City	N/A	1,532*	1,531	0.05
Cape Town	2454	N/A	2,451	0.12

*The area of Washington D.C. and Sao Paulo city were from the local governmental data online separately not from Kennedy's research data due to the different definition of the regions.

Firstly, we needed to make sure the city definition were the same with Kennedy's. As assumed that the same definition of one region can reflect the same area information to a certain extent. So we compared GADM area information with Kennedy's data in the same region to reconfirm we had the identical boundaries.

Table S4. Fractional estimation of emission from electricity sector

City	Industrial Processes (tCO_2/cap)	Energy (tCO_2/cap)	Electricity/Energy (%)	Large Point Source (tCO_2/cap)	In-boundary FFE (tCO_2/cap)	ODIAC FFE(tCO_2/cap)	Differences (%)
Delhi	0	1.3	45.0	0.25	0.97	0.90	-7
Delhi(Max)	0	1.3	44.3	0.25	0.97	0.90	-7
Delhi(Min)	0	1.3	36.8	0.25	1.07	0.90	-16
Tokyo	0.1	4.4	36.0	0.23	3.15	3.82	21
Tokyo(Max)	0.1	4.4	39.7	0.23	2.98	3.82	28
Tokyo(Min)	0.1	4.4	25.2	0.23	3.62	3.82	5
Washington D.C.	0	18	35.0	0.21	11.91	6.91	-42
Washington D.C. (Max)	0	18	39.2	0.21	11.15	6.91	-38
Washington D.C. (Min)	0	18	25.2	0.21	13.67	6.91	-49

In absence of detailed data, Delhi, Tokyo and Washington D.C. only have emissions from the total energy sector (including electricity, heating & industrial fuels and ground transportation) with the exception of aviation and marine available: for these three cities assumptions were necessary to estimate the fraction of emission from electricity sector. We empirically estimated the fractions by referring to cities with similar geographical location and social economic development level. These fractional estimations contribute to sources of uncertainties in inventory-based in-boundary emissions for these three cities.

We chose the value of 45% for proportion of electricity in energy for Delhi based on the main coal supply for electricity generation [3] and similar densely populated megacity plagued by poor air quality in the Asian region, such as Beijing (40.7%) and Shanghai (44.3%). For Washington D.C., we assumed 35% as the proportion, a value in between emission percentages for the other two U.S. cities studied, New York City (39.2%) and Los Angeles (28.1%). Similarly for Tokyo, 36% of CO_2 emissions from energy contributed to electricity sector emission was assumed based on the average of some megacities in developed countries such as New York City (39.2%), Los Angeles (28.1%) and Greater London (39.7%), (Supplementary Table S1). These values should be treated with caution as actual proportion values may easily differ from our simple assumptions by 10% or more.

To evaluate the uncertainties caused by fraction estimation of emission from electricity sector, we calculated the in-boundary CO_2 emissions based on the maximum and minimum proportion of electricity in energy for Delhi, Tokyo and Washington D.C. separately. Note this uncertainty range is only meant to assess the extent to which our assumption on fractional emission from electricity sector affects total in-boundary emission for the three cities, not to provide an absolute uncertainty range as the three cities could potentially deviate outside the range based on similar cities. for Delhi, we used the electricity fraction of 44.3% in Shanghai as the maximum proportion (note this is actually slightly smaller than the value we use for Delhi) of electricity and 36.8% in Bangkok as the minimum in the same Asian developing regions (including cities: Bangkok, Beijing, and Shanghai) with dense population and relatively poor air quality; for Washington D.C., we chose the maximum proportion of 39.2% for electricity in energy in New York City and the minimum proportion of 25.2% in Greater Toronto in terms of the similar economic development and energy consumption culture in North America (including cities: Los Angeles, New York City, and Greater Toronto); for Tokyo, according to the similar energy structure, role of city and economic development in developed countries (including cities: Greater London, New York City, Los Angeles, and Greater Toronto), we adopted the maximum proportion of 39.7% for electricity in energy in Greater London and the minimum proportion of 25.2% in Greater Toronto. Then maximum and minimum proportions for electricity in energy were multiplied total energy emissions and were subtracted from energy emissions. The remaining parts of energy emissions refer to the emissions from heating & industrial fuels and ground transportation, which were added to the large point sources and industrial processes emissions to establish the relevant

in-boundary CO_2 emissions. The corresponding in-boundary CO_2 emissions with maximum and minimum electricity proportion in energy and the differences between the two methods of city CO_2 emission for these three megacities are showed in Table S4 above.

Out of the three cities, we found without the uncertainty estimation that the difference between inventory-based CO_2 emission estimate and ODIAC estimate is within 10% for Delhi, within 30% for Tokyo, and over 30% for Washington D.C.. By applying the maximum/minimum electricity portion assumptions, the difference between the two methods of city in-boundary CO_2 emission estimates are 7-16% for Delhi, 5-28% for Tokyo, and 38-49% for Washington D.C..

Table S5. New York City population by Manhattan

Year	Bore share of NYC total (%)	Population
2000	19.20	1,537,195
2010	19.24	1,585,873
2005	19.22	1,570,274

The population of other 13 cities can be adopted the reported population data in literature directly [1, 4] directly except Manhattan. In absence of population data in Manhattan, we used the open New York City population data by borough from NYC Open Data (https://opendata.cityofnewyork.us/). The NYC Open Data reports the population and population proportion of New York City by borough ever decade from 1950 to 2040. So we simply assumed the bore share of New York City population by Manhattan in 2005 is the average of proportions in 2000 and 2010. Then the average population proportion of 19.22% was multiplied by the population of New York City in 2005 in Kennedy's research paper [4] to calculate the population of 1,570,274 for Manhattan in 2005.

Table S6. In-boundary fossil fuel emissions for cities and metropolitan regions

City	In-boundary FFE (tCO_2/cap)	Total in-boundary FFE($MtCO_2$)
Bangkok	4.86	27.53
Beijing	7.29	115.19
Shanghai	9.91	179.91
Delhi	0.96	12.72
Cape Town	1.75	6.12
Sao Paulo	0.94	10.58
Tokyo	3.15	39.94
Greater Paris	4.36	50.30
Greater London	4.39	32.36
Los Angeles	8.16	77.64
Manhattan	4.74	7.44
New York City	5.28	43.13
Washington D.C.	11.91	6.81
Greater Toronto	7.98	44.35

Table S7. Differences between calculations of emissions from ground transportation

City	Fuel sales (ML)	VKT-Fuel consumption(ML)	Scaled fuel consumption (ML)	Difference (%)
Bangkok	2741	2662	N/A	-2.9
Greater Toronto	6691	6988	N/A	4.4
New York City	N/A	4179	4107	-1.7

Emissions from ground transportation in Kennedy et al [5] were calculated by three methods based on local fuel sales within the city, vehicle kilometers travelled (VKT) in the boundary of the city, and scaling from wider regional data. The first method is to use local fuel sales multiplied by the IPCC emissions factors with default. For CO_2 emissions, this use of local fuel sales data is preferred by the IPCC and appropriate where the number of daily commuter trips across the city boundary is smaller than the number of trips within the city. The second approach based on gasoline consumptions estimated from VKT is appropriate for central cities where the number of cross-boundary commuter trips is large and the fuel sales may occur out of the central city boundary. This VKT for cities used computer models or by vehicle counting surveys differ between cities due to the different counting techniques and the unique model for each city. The third method is to scale gasoline consumptions from state, provincial, or wider regional data based on the consumption that vehicle in the city travel the same average annual kilometers as in the wider region. And the scaling factor may be determined by population corresponding to the total travel commute times. Although the third method is not desirable as other two approaches, this method is necessary when there is no reliable data in the city. In order to estimate the greatest source of uncertainty caused by ground transportation calculation, the first method was applied to Cape Town, Greater Toronto, and Bangkok; the second method was established for Bangkok, Greater London, and New York City; moreover, the third approach was used for Los Angeles, New York City, and Greater Toronto. Three of the cities, Greater Toronto, New York City, and Bangkok were applied by two different methods of these three separately. The comparisons between the calculations of these three approaches show that the differences can be less than 5% and these three methods can establish reasonably close estimates.

Table S8. Differences between calculations of emissions from DARTE and inventory -based data

City	Year (Kennedy)	Area (km2)	Ground Transportation(t CO2)	DARTE(MgCO2)	Difference (%)
Los Angeles	2000	10,612	46,835,143.0	42,842,342.9	9.3
New York City	2005	807	12,500,100.0	11,588,570.2	7.9
Washington D.C.	2000	178	3,922,019.8	1,667,169.2	135.3
Manhattan	2005	69	2,402,519.2	1,155,456.9	107.9

Table S9. Differences between CO2 emissions by electricity sector from eGRID and inventory -based data

City	Year(Kennedy)	Area (km2)	eGrid plant annual CO2 emissions (t CO2)	CARMA (t CO2)	Difference (%)
Los Angeles	2000	10,612	16,895,543.2	15,672,507.2	-7.2
New York City	2005	807	15,153,227.8	5,061,039.6	-66.6
Washington D.C.	2000	178	191,797.2	120,541.9	-37.2
Manhattan	2005	69	1,888,797.3	122,144.3	-93.5

References

[1] Kennedy C A, Ramaswami A, Carney S, and Dhakal S 2009b Greenhouse gas emission baselines for global cities and metropolitan regions In: Plenary paper presented at the World Bank's Fifth Urban Research Symposium

[2] Ramachandra T, Aithal B H and Sreejith K 2015 GHG footprint of major cities in India *Renewable and Sustainable Energy Reviews* **44** 473-495

[3] Mittal M L, Sharma C and Singh R 2012 Estimates of emissions from coal fired thermal power plants in India In: Proceedings of the 2012 International emission inventory conference

[4] Kennedy C A, Ibrahim N and Hoornweg D 2014 Low-carbon infrastructure strategies for cities *Nat. Clim. Change* **4** 343-346

[5] Kennedy C A *et al* 2010 Methodology for inventorying greenhouse gas emissions from global cities *Energ. Policy* **38** 4828-4837
